# Characteristics and risk factors of immune-related adverse events in patients receiving immune checkpoint inhibitor combination therapy

**DOI:** 10.3389/fonc.2026.1808832

**Published:** 2026-05-29

**Authors:** Yao Qiu, Xincai Zhao, Juan Chen, Manman Zhang, Yonggang Wang, Cheng Guo, Jianping Zhang, Rong Xu

**Affiliations:** 1 Department of Pharmacy, The Sixth People’s Hospital Affiliated to Shanghai Jiao Tong University Medical School, Shanghai, China; 2 Department of Oncology, The Sixth People’s Hospital Affiliated to Shanghai Jiao Tong University Medical School, Shanghai, China

**Keywords:** combination therapy, immune checkpoint inhibitors, immune-related adverse events, onset time, risk factors

## Abstract

**Objectives:**

To overcome the limitations of immune checkpoint inhibitor (ICI) monotherapy, combination strategies are increasingly adopted in clinical practice, which may further complicate the occurrence profile of immune-related adverse events (irAEs). This study aimed to investigate the characteristics, onset time, and risk factors of irAEs and endocrine-irAEs (EirAEs) in cancer patients receiving ICI combination therapy.

**Methods:**

Demographic data, treatment regimens, concomitant medications, and laboratory biomarkers were collected from 297 cancer patients, of whom 282 (94.95%) received ICI combination therapies. The incidence, severity, and onset time of irAEs were analyzed. Binary logistic regression was used to evaluate factors associated with irAEs and EirAEs, respectively. Model performance was assessed by the receiver operating characteristic (ROC) curve analysis.

**Results:**

IrAEs were seen in 113(38.05%) patients, with 4.38% experiencing grade≥3 irAEs. The most common irAEs were endocrine (12.12%), gastrointestinal (8.75%), and dermatological (6.73%) toxicities. For irAEs, ICI combined with chemotherapy accounted for the highest proportion (49.18%). For EirAEs, the proportion of ICI combined with targeted therapy was the highest(36.11%). Binary logistic regression analysis model revealed corticosteroid use was a protective factor for irAEs (OR = 0.511, 95%CI:0.296-0.884, P = 0.016), and age≥65 years was associated with lower risk (OR = 0.487, 95%CI:0.292-0.813, P = 0.006). Chemotherapy and targeted therapy did not show a significant impact on the overall incidence of irAEs. For EirAEs, combination with targeted therapy (OR = 2.888, 95%CI:1.186-7.033, P = 0.020) and higher baseline TSH levels (OR = 1.032, 95%CI:1.002-1.063, P = 0.038) were risk factors, while corticosteroid use was a protective factor(OR = 0.231, 95%CI:0.092-0.581, P = 0.002). The average time to onset of irAEs was 2.43 treatment cycles, with 85.25% emerging within 4 cycles, and endocrine toxicity exhibited delayed onset.

**Conclusion:**

Among patients predominantly treated with ICI combination therapy, irAEs were diverse and primarily low-grade, with the majority emerging within the first 4 treatment cycles. For irAEs, Age≥65 years was associated with lower risk, and corticosteroid use was a protective factor. For EirAEs, combination with targeted therapy and higher baseline TSH levels were risk factors, while corticosteroid use remained a protective factor. Onset time varied across different irAEs. These findings provide evidence to support risk-stratified monitoring and early intervention in clinical practice. Multicenter prospective studies are warranted for further validation.

## 1 Introduction

In recent years, immune checkpoint inhibitors (ICIs) have transformed the cancer treatment landscape. By targeting immunity downregulators such as cytotoxic T-lymphocyte-associated protein 4 (CTLA-4), programmed cell death protein-1 (PD-1) and programmed cell death ligand 1 (PD-L1) (1), ICIs can reactivate antitumor immunity, enabling the immune system to identify and eliminate tumor cells and leading to sustained immune responses (1–3). Their indications have rapidly expanded from advanced melanoma and non-small cell lung cancer to encompass a broad range of solid tumors, establishing a new standard of treatment (4, 5).

However, despite remarkable efficacy, ICIs are confronted with significant challenges, including primary and acquired resistance, heterogeneous response rates, and immune-related adverse events (irAEs) thought to result from reactivated cellular immunity (6–9). These toxicities occur because the reactivated immune response is not entirely tumor-specific and can affect nearly all organ systems, ranging from mild and self-limiting symptoms to life-threatening complications that require prompt recognition and management (2, 10). Common irAEs include dermatological toxicity, gastrointestinal toxicity, hepatic toxicity, and endocrine disturbances (including thyroid dysfunctions and hypophysitis) (11–13). The management of these events usually requires immunosuppression with corticosteroids or other immunomodulators, and in severe cases may lead to permanent treatment discontinuation (11, 14).

To overcome the limitations of ICI monotherapy, combination therapy (CT) has emerged as a promising approach and is widely used in clinical practice. Common regimens include the combination with chemotherapy, targeted therapy, and other ICIs. The rationale behind CT lies in the acknowledgment that cancers are inherently multifactorial and thus unlikely to be effectively controlled by single-pathway interventions alone. By targeting distinct biological pathways or mechanisms, CT holds the potential to synergistically augment therapeutic efficacy while alleviating adverse effects and slowing down the emergence of drug resistance (15, 16). The combination of ICIs with chemotherapy has demonstrated particularly notable clinical success. Chemotherapy can interact with different components of the immune system, enhancing immunogenicity while suppressing immune-suppressive functions within the tumor microenvironment (TME). Some chemotherapeutic agents enhance tumor infiltration and boost the activity of effector cells, such as cytotoxic T lymphocytes, dendritic cells(DCs), and natural killer cells (15, 17, 18). Targeted therapies can induce rapid tumor regressions, with a consequent decrease in tumor-associated immunosuppression, and they may afford a favorable window for immunotherapy to achieve more potent cytotoxicity. Moreover, the release of large amounts of antigenic debris may contribute to vaccination *in situ*, particularly if concurrent DC activation can be triggered (19). Anti-angiogenic drugs can reverse immune suppression by increasing intra-tumoral effector cells, decreasing PD-L1 expression, and reducing infiltrating myeloid-derived suppressor cells (MDSCs) and regulatory T cells (Tregs).

Studies have shown that combining chemotherapy with PD-1 inhibitors or combining CTLA-4 inhibitors with PD-1 or PD-L1 inhibitors, does not lead to unexpected new irAEs and the reported adverse effects are consistent with those of each agent alone. However, the frequency of adverse events with combination therapy is higher than with monotherapy (20–24). Furthermore, combination therapy poses significant diagnostic and therapeutic challenges in clinical practice, as typical side effects of cytotoxic chemotherapy overlap with the inflammatory manifestations of irAEs, yet their management strategies are fundamentally different. Currently, there are no available biomarkers in daily routine that help distinguish cytotoxic side effects from irAEs, and moreover, there are no available recommendations to manage patients undergoing treatment with a combination of chemotherapy and immunotherapy (25, 26).

The incidence and spectrum of irAEs are influenced by multiple factors. Emerging evidence indicates that irAEs not only vary among different drug classes (for instance, anti-CTLA-4 inhibitors are associated with a higher incidence of irAEs compared to anti-PD-1/PD-L1 inhibitors), but also vary among different drugs acting on the same target (27–29). Additionally, patient-specific factors, such as a history of autoimmune diseases, concomitant medications (e.g., antibiotics, proton-pump inhibitors), and biomarkers like the neutrophil-to-lymphocyte ratio (NLR), are all associated with changes in the risk of developing irAEs (1, 30). Research on predictive biomarkers for irAEs remains incomplete. Most existing data derived from strictly selected clinical trial populations, and real-world studies on laboratory parameters and concomitant medications are limited and show inconsistent results (1, 30, 31). There is an urgent need to gather evidence from real-world patient cohorts in which combination therapy has become routine, in order to better understand its safety profile, risk factors, and the influence of different combination regimens on irAE patterns, thereby optimizing management strategies (26).

In this context, this study aims to characterize the real-world incidence, types, onset time and risk factors of irAEs among patients predominantly treated with ICI combination therapy. These findings can assist in providing evidence to support risk-stratified monitoring and early intervention in clinical practice.

## 2 Methods

### 2.1 Study population

This study retrospectively analyzed cancer patients treated with ICIs at a tertiary general hospital between January 2023 and December 2024. Eligible participants included patients with clinically and pathologically confirmed solid malignancies who received ICI-based therapies. The study excluded patients with more than 20% missing baseline variables, those participating in clinical trials, and individuals under 18 years of age. This study was approved by the Ethics Committee of Shanghai Sixth People’s Hospital (Approval number: 2025-KY-474(K)), and the need for informed consent was waived because of the retrospective nature of this study.

### 2.2 Data collection

Data were obtained from the hospital information system, comprehensively covering the following: demographic information (age, sex, smoking history, height, weight, comorbidities), cancer types and TNM stage, ICIs, concomitant medications (defined as drugs used within 1 month before ICI therapy, including corticosteroids, proton-pump inhibitors(PPI), non-steroidal anti-inflammatory drugs(NSAIDs), and antibiotics), and laboratory biomarkers (baseline: interleukin-6 (IL-6), interleukin-8 (IL-8), tumor necrosis factor-alpha (TNF-α), triiodothyronine (T3), thyroxine (T4), thyroid-stimulating hormone (TSH), and pro-brain natriuretic peptide (proBNP), estimated glomerular filtration rate(eGFR); post-treatment: lactate dehydrogenase(LDH), NLR).

### 2.3 Study assessment

The causal relationship between ICIs and irAEs was assessed using the World Health Organization causality assessment, categorized as follows: “certain”, “probable”, “possible”, “unlikely”, “unclassified”, or “unclassifiable” (32). We evaluated and included those classified as “certain,” “probable,” or “possible” in the study. The severity of irAEs was graded according to the Common Terminology Criteria for Adverse Events (CTCAE) version 5.0 (33).The onset time of irAEs was defined as the interval between initiating ICI therapy and the observation of abnormal clinical, imaging, or laboratory results.

### 2.4 Statistical analysis

Continuous variables were reported as median and interquartile range (IQR), while categorical variables were expressed as frequency (percentage) N (%). For comparisons between groups, categorical variables were analyzed using the Chi-square or Fisher’s exact test, and continuous variables were compared using the Mann-Whitney U test (non-normal distribution). Variables with P<0.05 in univariate analysis were included in binary logistic regression analysis. According to the 10 Events Per Variable (EPV) principle (34, 35), to ensure model accuracy while avoiding overfitting, variables with P<0.05 in the univariate analysis of endocrine-irAEs (EirAEs) were further screened using Lasso regression (with λ1se selected as the optimal value) (36, 37). To assess the potential impact of confounders, sensitivity analysis was conducted by adding demographic variables to the binary logistic regression model. To maximize statistical power and minimize bias from excluding missing data, the MICE package was used to perform multiple imputation on variables with missing data, generating 50 imputed datasets. For missing continuous variables, predictive mean matching (pmm) was applied for imputation (**Supplementary Table 1**). Sensitivity analysis of logistic regression results was conducted using the complete dataset. Model performance was assessed by the receiver operating characteristic (ROC) curve analysis for discrimination accuracy. (An AUC value >0.5 in ROC analysis suggests satisfactory discriminatory ability of the model (38)). All statistical analyses were performed using IBM SPSS Statistics (version 25.0) and R statistical software (version 4.3.3). Visualizations were created with GraphPad Prism (version 8.0.2). P values were calculated based on two-sided hypotheses, with P<0.05 considered statistically significant.

## 3 Results

### 3.1 Clinical characteristics of patients

This study included 297 cancer patients treated with ICIs. **Table 1** details the clinical characteristics. There were 169 male patients (56.90%) and 128 female patients (43.10%). The median age was 60 years (IQR:51-69), with 120 patients (40.40%) aged≥65 years. The median body mass index(BMI) was 22.83 kg/m² (IQR:20.06-24.95). Comorbidities mainly included hypertension in 75 patients (25.25%), diabetes in 34 (11.45%), and hepatitis B history in 19 (6.40%). Among the patients, 282 (94.95%) received combination therapy: 155 (52.19%) received ICI combined with chemotherapy (I+C), 81 (27.27%) received ICI combined with chemotherapy and targeted therapy (I+C+T), and 46 (15.49%) received ICI combined with targeted therapy (I+T).

**Table 1:** Characteristics of patients (N = 297).

Characteristics	N (%)	Characteristics	N (%)
Sex		Colorectal cancer	17 (5.72%)
Male	169 (56.90%)	Head and neck cancer	15 (5.05%)
Female	128 (43.10%)	Esophageal cancer	10 (3.37%)
Age		Other cancers	30 (10.10%)
Median (IQR)	60 (51, 69)	ICIs	
<65	177 (59.60%)	PD-1 inhibitor	268 (90.24%)
≥65	120 (40.40%)	Sintilimab	129 (43.43%)
BMI, kg/m²	22.83 (20.06, 24.95)	Tislelizumab	76 (25.59%)
Smoking history		Camrelizumab	40 (13.47%)
No	248 (83.50%)	Toripalimab	13 (4.38%)
Yes	49 (16.50%)	Serplulimab	6 (2.02%)
Comorbidities		Pembrolizumab	3 (1.01%)
No	182 (61.28%)	Penpulimab	1 (0.34%)
Yes	115 (38.72%)	PD-L1 inhibitor	25 (8.42%)
High pressure		Adebrelimab	21 (7.07%)
No	222 (74.75%)	Atezolizumab	3 (1.01%)
Yes	75 (25.25%)	Envafolimab	1 (0.34%)
Diabetes		PD-1/CTLA-4 inhibitor	4 (1.35%)
No	263 (88.55%)	Cadonilimab	4 (1.35%)
Yes	34 (11.45%)	eGFR<60mL/min/1.73m²	
Heart disease		No	279 (93.94%)
No	279 (93.94%)	Yes	18 (6.06%)
Yes	18 (6.06%)	Corticosteroids	
Lung disease		No	108 (36.36%)
No	290 (97.64%)	Yes	189 (63.64%)
Yes	7 (2.36%)	NSAIDs	
Hepatitis B		No	224 (75.42%)
No	278 (93.60%)	Yes	73 (24.58%)
Yes	19 (6.40%)	Antibiotics	
Cancer stage		No	246 (82.83%)
I-III	62 (20.88%)	Yes	51 (17.17%)
IV	235 (79.12%)	PPI	
Treatment approaches		No	215 (72.39%)
I+C	155 (52.19%)	Yes	82 (27.61%)
I+C+T	81 (27.27%)	Laboratory biomarkers	
I+T	46 (15.49%)	LDH, U/L	200.50 (163.50, 252.75)
I	15 (5.05%)	IL-6, pg/mL	9.70 (5.22, 22.43)
Cancer types		IL-8, pg/mL	19.25 (10.38, 40.15)
Stomach cancer	61 (20.54%)	TNF-α,pg/ml	2.00 (1.00, 4.20)
Lung cancer	47 (15.82%)	NLR	3.56 (1.87, 6.10)
Gynecological cancer	39 (13.13%)	T3, pmol/L	4.32 (3.77, 4.82)
Soft tissue cancer	37 (12.46%)	T4, pmol/L	15.50 (14.10, 17.38)
Bone cancer	24 (8.08%)	TSH, mIU/L	2.24 (1.43, 3.66)
Urinary system cancer	17 (5.72%)	proBNP, ng/L	77.25 (37.23, 142.25)

BMI, body mass index; ICIs, immune checkpoint inhibitors; I, C and T, immunotherapy, chemotherapy, targeted therapy; PD-1, programmed cell death protein-1; PD-L1, programmed cell death ligand 1; CTLA-4, cytotoxic T-lymphocyte-associated protein 4; eGFR, estimated glomerular filtration rate; PPI, proton-pump inhibitors; NSAIDs, non-steroidal anti-inflammatory drugs; LDH, lactate dehydrogenase; IL-6, interleukin-6; IL-8, interleukin-8; TNF-α, tumor necrosis factor-alpha; T3, triiodothyronine; T4, thyroxine; TSH, thyroid-stimulating hormone; NLR, neutrophil-to-lymphocyte ratio; proBNP, pro-brain natriuretic peptide.

The most common tumor types were stomach cancers with 61 patients (20.54%), followed by lung cancers with 47 patients (15.82%). 235 patients (79.12%) were in stage IV. 268 patients (90.24%) were treated with PD-1 inhibitors, mainly Sintilimab, Tislelizumab, and Camrelizumab.

Concomitant medications included corticosteroids in 189 patients (63.64%), NSAIDs in 73 (24.58%), antibiotics in 51 (17.17%), and PPIs in 82 (27.61%).

### 3.2 Characteristics of irAEs

Among the 297 patients, 113 patients (38.05%) experienced irAEs, with a total of 122 events. Of these, 13 patients (4.38%) experienced severe irAEs of grade≥3.The distribution of different types of irAEs (**Table 2**) showed that the most common irAEs were endocrine toxicity(12.12%), gastrointestinal toxicity(8.75%), dermatologic toxicity(6.73%), hepatic toxicity(4.71%), and hematological toxicity (3.70%). The majority of grade≥3 irAEs were observed in dermatologic toxicity (5 cases) and hematological toxicity (4 cases). Although endocrine toxicity had a relatively higher incidence, all EirAEs were grade 1-2.

**Table 2:** Characteristics of irAEs (N = 297).

Types of irAEs	Grade 1	Grade 2	Grade 3	Grade 4	N (%)
Endocrine toxicity	19	17	0	0	36 (12.12%)
Gastrointestinal Toxicity	16	9	1	0	26 (8.75%)
Dermatologic Toxicity	5	10	5	0	20 (6.73%)
Hepatic toxicity	9	4	1	0	14 (4.71%)
Hematologic Toxicity	5	2	2	2	11 (3.70%)
Neurologic toxicity	3	3	0	0	6 (2.02%)
Cardiac toxicity	2	1	0	1	4 (1.35%)
Lung toxicity	2	0	1	0	3 (1.01%)
Renal toxicity	1	1	0	0	2 (0.67%)

irAEs, immune-related adverse events. The severity grouping, ranging from Grade1 to Grade5. Grade1 indicates less severe adverse events, and Grade 5 indicates the most severe. No Grade 5 adverse events were observed.

**Figure 1:**
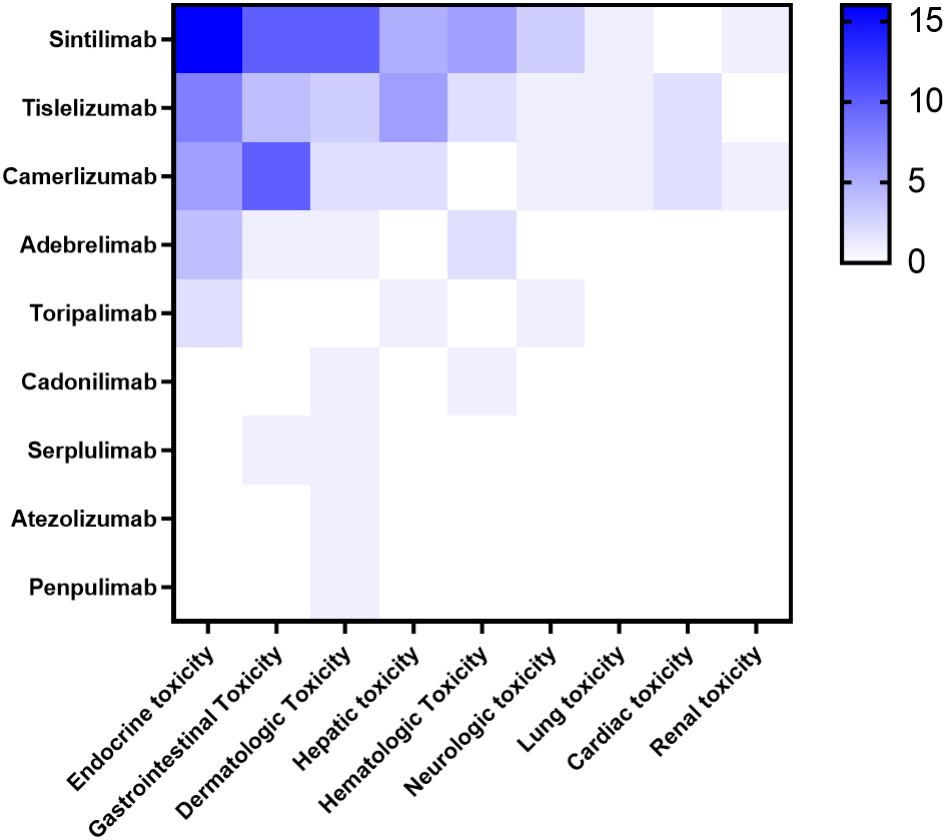
Distribution of irAEs among the ICIs. irAEs, immune-related adverse events; ICIs, immune checkpoint inhibitors. Darker color indicates a higher reported number of irAEs for that ICI in the specific toxicity.

The distribution of irAEs varied across different ICIs (**Figure 1**; **Supplementary Table 2**). Endocrine toxicity was more commonly seen in Sintilimab (16 cases, 44.44%) and Tislelizumab (8 cases, 22.22%). Gastrointestinal toxicity was more common in Sintilimab (10 cases, 38.46%) and Camrelizumab (10 cases, 38.46%). Dermatologic toxicity was observed across various types of ICIs.

The incidence of irAEs differed among treatment approaches. The highest incidence of irAEs was observed in the I+T group (24/46, 52.17%), followed by I group (6/15, 40.00%), I+C+T group (31/81, 38.27%), and I+C group (52/155, 33.55%). The high incidence of irAEs in the I+T group may primarily be attributable to the high proportion of EirAEs (n=13). Regarding the distribution of irAEs, the results (**Table 3**) showed that most widely used I+C approaches accounted for 60 events (49.18%), I+C+T for 32 events (26.23%), I+T for 24 events (19.67%), and I for 6 events (4.92%). Regarding EirAEs, the results (**Table 4**) showed that I+T accounted for 13 events (36.11%), I+C+T and I+C each for 10 events (27.78%), and I for 3 events (8.33%).

**Table 3:** Characteristics of irAEs in different treatment approaches(N = 122).

ICIs	Sintilimab	Tislelizumab	Camerlizumab	Adebrelimab	Toripalimab	Cadonilimab	Serplulimab	Atezolizumab	Penpulimab	N(%)
I+C	25	9	19	3	1	0	1	1	1	60 (49.18%)
I+C+T	13	10	2	3	1	2	1	0	0	32 (26.23%)
I+T	12	4	4	2	2	0	0	0	0	24 (19.67%)
I	2	4	0	0	0	0	0	0	0	6 (4.92%)

irAEs, immune-related adverse events; I, C and T, immunotherapy, chemotherapy, targeted therapy.

**Table 4:** Characteristics of EirAEs in different treatment approaches (N = 36).

ICIs	Sintilimab	Tislelizumab	Camerlizumab	Adebrelimab	Toripalimab	N(%)
I+T	7	1	2	2	1	13 (36.11%)
I+C	4	1	4	1	0	10 (27.78%)
I+C+T	4	4	0	1	1	10 (27.78%)
I	1	2	0	0	0	3 (8.33%)

EirAEs, Endocrine immune-related adverse events; I, C and T, immunotherapy, chemotherapy, targeted therapy.

The distribution of irAEs and EirAEs across different cancer types was analyzed. As shown in **Supplementary Table 3**, the highest irAE incidence was observed in colorectal cancer (9/17, 52.94%), followed by bone cancer (10/24, 41.67%), and urinary system cancer (7/17, 41.18%). Esophageal cancer showed the lowest irAE incidence (1/10, 10.00%). Regarding EirAEs, soft tissue cancer (8/37, 21.62%), bone cancer (5/24, 20.83%), and head and neck cancer (3/15, 20.00%) exhibited relatively higher rates of endocrine toxicity, while lung cancer (1/47, 2.13%) and colorectal cancer (1/17, 5.88%) showed lower rates.

### 3.3 Onset time of irAEs

Analysis of irAE onset time (**Table 5**) showed that 104 events(85.25%) occurred within 4 treatment cycles following the administration of ICIs, and the average time to irAE occurrence was 2.43 cycles. Hepatic toxicity appeared relatively early (average 1.43 cycles). Following this, renal, hematological, gastrointestinal, dermatologic, neurological, cardiac, and lung toxicities occurred (average 2.00-3.00 cycles). Renal and lung toxicities had low incidences, and their onset time required further validation in subsequent studies. Endocrine toxicity had an average onset time of 3.39 cycles, with over 30% of events occurring after the 4th cycle, indicating significant delay and persistence.

**Table 5:** Time to adverse events after treatment with ICIs.

irAEs, N (%)	Course 1	Course 2	Course 3	Course 4	Course>4	Average course of occurrence
Endocrine Toxicity	17 (47.22%)	5 (13.89%)	3 (8.33%)	0 (0.00%)	11 (30.56%)	3.39
Lung Toxicity	2 (66.67%)	0 (0.00%)	0 (0.00%)	0 (0.00%)	1 (33.33%)	3.00
Cardiac Toxicity	2 (50.00%)	0 (0.00%)	1 (25.00%)	0 (0.00%)	1 (25.00%)	2.50
Neurologic Toxicity	2 (33.33%)	2 (33.33%)	1 (16.67%)	0 (0.00%)	1 (16.67%)	2.50
Dermatologic Toxicity	8 (40.00%)	4 (20.00%)	5 (25.00%)	2 (10.00%)	1 (5.00%)	2.35
Gastrointestinal Toxicity	16 (61.54%)	5 (19.23%)	2 (7.69%)	1 (3.85%)	2 (7.69%)	2.12
Hematologic Toxicity	8 (72.73%)	1 (9.09%)	1 (9.09%)	0 (0.00%)	1 (9.09%)	2.00
Renal Toxicity	1 (50.00%)	0 (0.00%)	1 (50.00%)	0 (0.00%)	0 (0.00%)	2.00
Hepatic Toxicity	9 (64.29%)	4 (28.57%)	1 (7.14%)	0 (0.00%)	0 (0.00%)	1.43

ICIs, immune checkpoint inhibitors; irAEs, immune-related adverse events.

### 3.4 Analysis of risk factors

**Figure 2:**
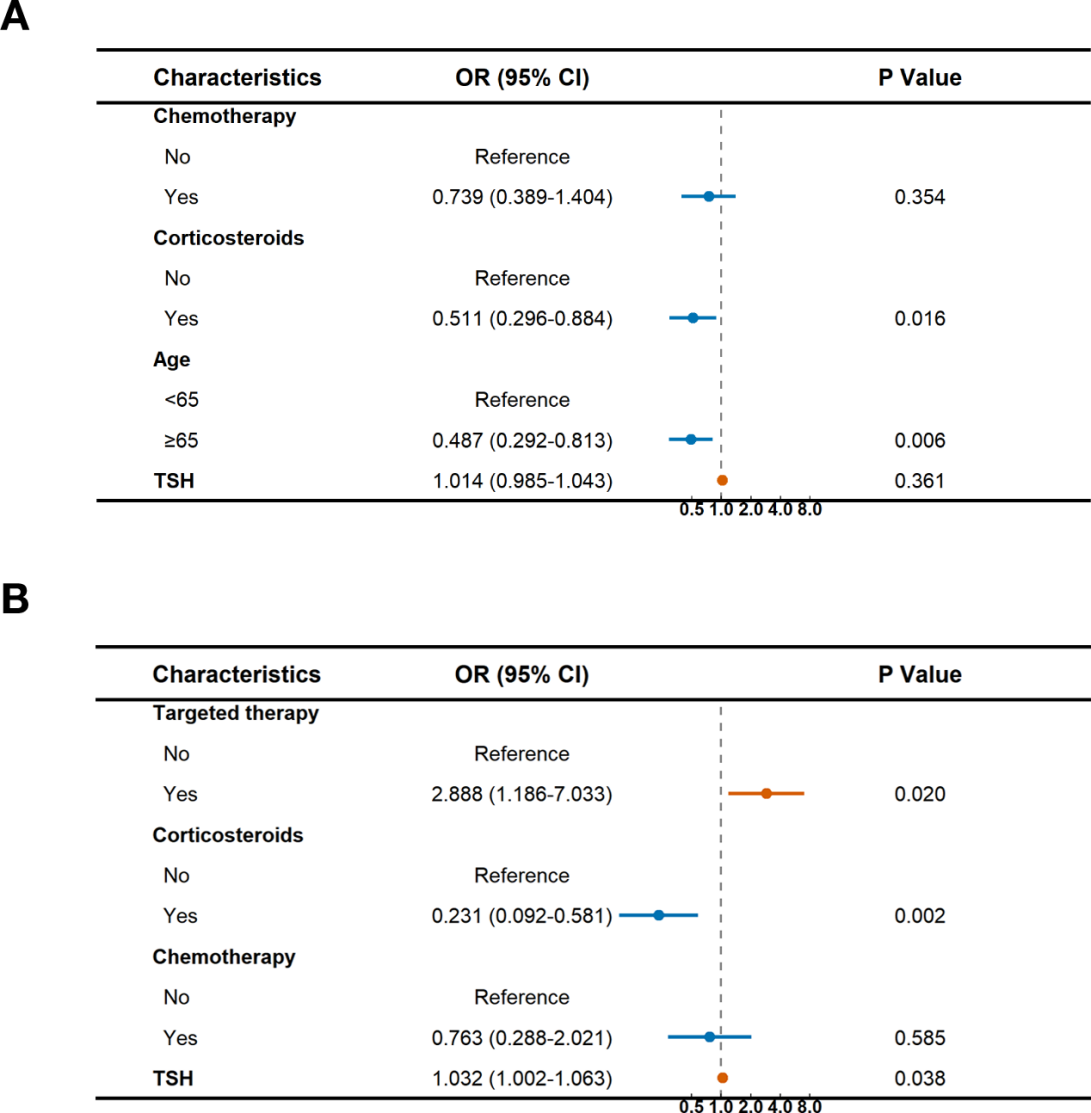
Binary logistic regression analysis to determine risk factors for incidence of irAEs and EirAEs. **(A)** irAEs. **(B)** EirAEs. irAEs, immune-related adverse events; EirAEs, endocrine immune-related adverse events.

The univariate analysis of risk factors for irAEs (**Table 6**) revealed significant differences between the irAEs group and the non-irAEs group in terms of age, use of corticosteroids, combination with chemotherapy, and baseline TSH levels (P<0.05). Binary logistic regression analysis (**Figure 2A**) further indicated that the use of corticosteroids (OR = 0.511, 95%CI:0.296-0.884, P = 0.016) was a protective factor against irAEs. Age≥65 years was associated with a significantly lower risk of irAEs compared to age<65 years (OR = 0.487, 95%CI:0.292-0.813, P = 0.006). Combination with chemotherapy and baseline TSH levels did not significantly correlate with risk of irAEs.

**Table 6:** Univariate analysis to determine risk factors for incidence of irAEs and EirAEs.

Characteristics	Non-irAEs (n=184)	irAEs (n=113)	χ²/z value	P Value	Non-EirAEs (n=261)	EirAEs (n=36)	χ²/z value	P Value
Sex			0.308	0.579			1.565	0.211
Male	107	62			152	17		
Female	77	51			109	19		
Age			8.060	0.005			4.037	0.045
<65	98	79			150	27		
≥65	86	34			111	9		
Smoking history			0.280	0.597			0.202	0.653
No	152	96			217	31		
Yes	32	17			44	5		
Comorbidities			0.457	0.499			0.118	0.732
No	110	72			159	23		
Yes	74	41			102	13		
High pressure			0.486	0.486			0.732	0.392
No	135	87			193	29		
Yes	49	26			68	7		
Diabetes			0.528	0.467			0.241	0.581
No	161	102			232	31		
Yes	23	11			29	5		
Heart disease			3.716	0.054			0.775	0.707
No	169	110			244	35		
Yes	15	3			17	1		
Lung disease			1.109	0.433			1.821	0.203
No	181	109			256	34		
Yes	3	4			5	2		
Hepatitis B			0.013	0.911			0.256	0.713
No	172	106			245	33		
Yes	12	7			16	3		
Cancer stage			0.015	0.904			0.422	0.516
I-III	38	24			53	9		
IV	146	89			208	27		
Chemotherapy			4.037	0.045			14.345	<0.001
No	31	30			45	16		
Yes	153	83			216	20		
Targeted therapy			2.604	0.107			7.471	0.006
No	112	58			157	13		
Yes	72	55			104	23		
Cancer types			7.117	0.625			15.371	0.081
Stomach cancer	37	24			56	5		
Lung cancer	31	16			46	1		
Gynecological cancer	23	16			35	4		
Soft tissue cancer	26	11			29	8		
Bone cancer	14	10			19	5		
Urinary system cancer	10	7			14	3		
Colorectal cancer	8	9			16	1		
Head and neck cancer	9	6			12	3		
Esophageal cancer	9	1			10	0		
Other cancers	17	13			24	6		
ICIs			0.463	0.879			0.645	0.718
PD-1 inhibitor	166	102			236	32		
PD-L1 inhibitor	16	9			21	4		
PD-1/CTLA-4 inhibitor	2	2			4	0		
eGFR<60mL/min/1.73m²			0.181	0.671			0.018	1.000
No	172	107			245	34		
Yes	12	6			16	2		
Corticosteroids			10.287	0.001			19.373	<0.001
No	54	54			83	25		
Yes	130	59			178	11		
NSAIDs			2.569	0.109			1.384	0.240
No	133	91			194	30		
Yes	51	22			67	6		
Antibiotics			1.164	0.281			0.310	0.577
No	149	97			215	31		
Yes	35	16			46	5		
PPI			0.731	0.392			0.140	0.709
No	130	85			188	27		
Yes	54	28			73	9		
BMI, kg/m²	22.5 (19.8,24.9)	23.1 (20.4,25.3)	-1.500	0.134	22.8 (20.0,25.0)	23.2 (20.6,24.4)	-0.561	0.576
LDH, U/L	193 (162,237)	209 (168,273)	-1.790	0.073	199 (163,251)	190 (163,243)	0.315	0.720
IL-6, pg/mL	10.30 (5.51,23.50)	8.96 (4.98,18.60)	0.952	0.342	9.70 (5.00,20.80)	8.50 (6.02,22.60)	-0.489	0.626
IL-8, pg/mL	19.8 (10.5,46.2)	17.2 (10.5,32.6)	0.695	0.487	18.8 (10.2,39.6)	20.7 (11.0,39.8)	-0.524	0.601
TNF-α, pg/ml	2.13 (1.17,4.37)	1.83 (0.82,4.29)	1.210	0.228	2.00 (1.03,4.50)	2.24 (0.98,3.76)	-0.142	0.848
NLR	3.79 (1.93,6.20)	3.21 (1.84,5.60)	0.794	0.428	3.88 (2.01,6.32)	2.17 (1.67,3.14)	3.540	**<0.001**
T3, pmol/L	4.27 (3.78,4.72)	4.39 (3.72,4.85)	-0.716	0.474	4.32 (3.81,4.78)	4.18 (3.60,4.86)	0.286	0.768
T4, pmol/L	15.5 (14.0,17.2)	15.5 (14.3,17.6)	-0.605	0.545	15.7 (14.1,17.5)	14.9 (13.5,16.6)	1.760	0.078
TSH, mIU/L	2.12 (1.39,3.32)	2.53 (1.64,4.75)	-2.190	**0.029**	2.16 (1.40,3.45)	3.63 (2.14,7.79)	-3.470	**<0.001**
proBNP, ng/L	79.1 (39.4,163.0)	65.8 (33.4,128.0)	1.340	0.179	74.4 (38.4,149.0)	69.0 (30.2,127.0)	0.943	0.346

irAEs, immune-related adverse events; EirAEs, Endocrine immune-related adverse events. Categorical variables were compared using the χ² test or Fisher exact test, and continuous variables using the Mann-Whitney U test (z value reported). Significant values are in bold.

The univariate analysis of risk factors for EirAEs (**Table 6**) showed significant differences between the EirAEs group and the non-EirAEs group in terms of age, combination with targeted therapy, combination with chemotherapy, use of corticosteroids, NLR, and baseline TSH levels (P<0.05). Building on the univariate screening results from the Lasso regression(**Supplementary Table 4**), four independent variables were ultimately included in the binary logistic regression model of EirAEs. Binary logistic regression analysis (**Figure 2B**) further demonstrated that combination with targeted therapy (OR = 2.888, 95%CI:1.186-7.033, P = 0.020) and higher baseline TSH levels (OR = 1.032, 95%CI:1.002-1.063, P = 0.038) were risk factors for EirAEs, while the use of corticosteroids was a protective factor (OR = 0.231, 95%CI:0.092-0.581, P = 0.002).No significant interaction was observed between combination with targeted therapy and combination with chemotherapy(P = 0.846).

### 3.5 Model validation

**Figure 3:**
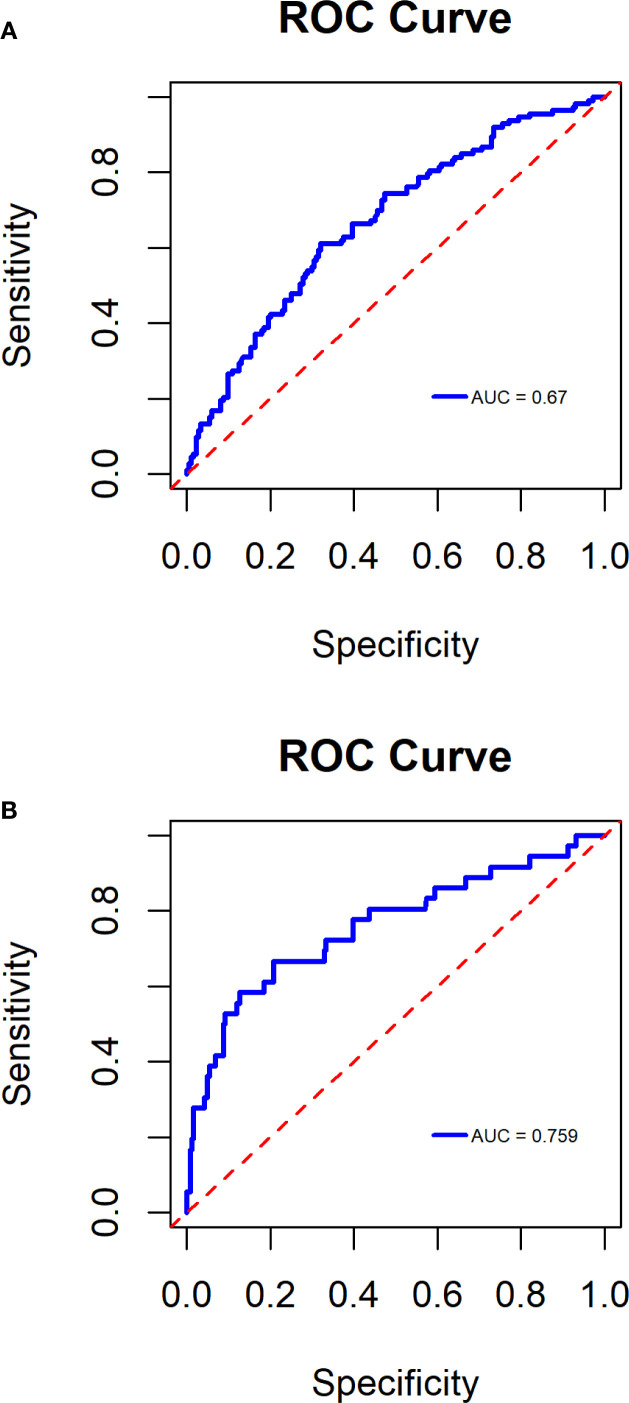
ROC curve of the logistic regression model for irAEs and EirAEs. **(A)** irAEs. **(B)** EirAEs. ROC, receiver operating characteristic; irAEs, immune-related adverse events; EirAEs, Endocrine immune-related adverse events; AUC, area under the curve. AUC (irAEs) = 0.670, 95%Cl: 0.607-0.730, AUC (EirAEs) = 0.759, 95%CI: 0.665-0.846.

To evaluate the performance of the logistic regression models, we conducted validation using ROC curve analysis. ROC curve analysis (**Figure 3**) showed an area under the curve (AUC) of 0.670 (95%CI: 0.607-0.730) for the irAEs model and 0.759 (95%CI: 0.665-0.846) for the EirAEs model, indicating acceptable and good discriminative ability, respectively.

### 3.6 Sensitivity analysis

The sensitivity analysis of the binary logistic regression for irAEs and EirAEs (**Supplementary Tables 5**, **S6**) showed that, despite the inclusion of demographic variables, sex, smoking history, BMI, and comorbidities, the results remained largely consistent with those before adjustment, demonstrating the robustness of the findings.

Both the irAEs and EirAEs complete datasets comprised 260 patients (87.54%). The results of binary logistic regression analysis using the complete dataset showed good consistency with those from imputed dataset (**Supplementary Tables 7**, **S8**), indicating that the primary analysis conclusions are robust to the missing data mechanism. Regarding irAEs, the use of corticosteroids was a significant protective factor in both datasets, and age≥65 years was associated with a lower risk of irAEs compared to age<65 years. Regarding EirAEs, the use of corticosteroids was a stable protective factor, while higher baseline TSH levels were a stable risk factor. Combination with targeted therapy showed a greater risk in the complete dataset (imputed dataset: OR = 2.888, 95%CI:1.186-7.033, P = 0.020; complete dataset: OR = 4.322, 95%CI:1.695-11.873, P = 0.003). Given the superior statistical power, the results from the imputed dataset were reported.

## 4 Discussion

### 4.1 Characteristics of irAEs

In this real-world cohort predominantly treated with combination therapy, the overall incidence of irAEs was 38.05%, and that of grade≥3 irAEs was 4.38%. The reported occurrence rate of irAEs varies substantially across studies (25.4%–55.9%) (31, 39–41). This discrepancy may stem from differences in study design, population included, and ICI selection. The use of combination strategies may enhance the efficacy of cancer immunotherapy but can also amplify irAEs (42–44). The incidences of grade≥3 irAEs in recently published two real-world studies were 4.6% and 5% (31, 45).

Endocrine, gastrointestinal, and dermatologic toxicities were the most common organ-specific irAEs, consistent with findings generally reported in the literature (12, 46). The ICI-mediated abrogation of checkpoint pathways is often associated with the development of immune-mediated inflammatory toxicities (43, 47), with the skin, gut, liver, and endocrine organs being among the most affected (48). EirAEs were predominantly characterized by thyroid dysfunction (hypothyroidism and hyperthyroidism), all of which were grade 1-2. A retrospective study on thyroid dysfunction in advanced lung cancer also reported only grade 1–2 adverse events (49). Another retrospective study of 2523 patients reported that 96.86% of EirAEs were grade 1–2 adverse events (41).

This study observed different toxicity profiles among ICIs. Endocrine toxicity was more frequently seen with Sintilimab and Tislelizumab. Gastrointestinal toxicity was more common with Sintilimab and Camrelizumab. Studies have shown that even drugs acting on the same target may have different occurrences of irAEs, and the same ICI can produce different toxicity profiles when applied to different tumors (29). A systematic review and meta-analysis pointed that while the overall average incidence of adverse events is similar across cancer types, differences exist between drugs. For example, Nivolumab was associated with a higher mean incidence of all-grade adverse events compared with Pembrolizumab(OR = 1.28, 95%CI:0.97-1.79) (50). The combination of immunotherapy with other agents such as chemotherapy or targeted therapy results in distinct toxicity patterns. For instance, Ipilimumab combined with dacarbazine leads to greater hepatotoxicity (51), while its combination with carboplatin and paclitaxel results in more frequent dermatologic manifestations (52).

### 4.2 Temporal patterns of irAEs

The average time to onset of all-grade irAEs in this study was 2.43 treatment cycles, with 85.25% occurring within the first 4 cycles. Hepatic toxicity emerged earliest, followed by hematologic, gastrointestinal, dermatologic, neurologic, cardiac, and lung toxicities, which typically presented around 1–2 cycles. In contrast, endocrine toxicities exhibited a delayed onset (average 3.39 cycles). A large retrospective study noted that most irAEs appeared within 1–4 cycles: initial presentations—including hematologic, ocular, and ototoxic events, as well as endocrine, hepatic, dermatologic, and gastrointestinal toxicities—usually occurred around 1–2 cycles after treatment initiation. Pneumonitis, skeletal muscle, neurologic, and renal toxicities tended to manifest after 2 cycles. Additionally, 20.7% of endocrine toxicities and 38.9% of cardiac toxicities showed delayed onset, emerging approximately 3 cycles later (41). IrAEs fall within the spectrum of autoimmune diseases and can occur at any time during ICI treatment. A pooled analysis of 23 clinical trials involving 8,436 patients reported that the median onset of most irAEs ranges from 2 to 15 weeks, with some events emerging as late as 6 months. Gastrointestinal, dermatologic, and hepatic toxicities are among the earliest irAE manifestations (53). Regarding hepatic toxicity onset, studies described cases occurring shortly after the first cycle of immunotherapy up to several months after its discontinuation (11, 54). Although hepatic toxicity had the earliest median onset in our study, the limited number of events warranted further validation. Other studies indicated that early irAEs most commonly presented as cutaneous toxicities, followed by gastrointestinal and hepatic events (1–2 months), endocrine toxicities (2–3 months), and renal toxicities (>3 months) (55, 56). Thyroid dysfunction is the most frequent ICI−associated endocrine adverse event (57). Typical thyroid toxicity often follows a biphasic pattern: transient thyrotoxicosis usually occurs within 2–6 weeks, followed by hypothyroidism around 12 weeks, which generally requires long−term levothyroxine replacement (58). This may explain the delayed onset observed in some patients with thyroid toxicity.

This temporal heterogeneity calls for dynamic, individualized monitoring protocols. During the early treatment phase (first 2 cycles), close monitoring of complete blood counts and liver function is essential to capture potential early immune-mediated injury. For thyroid function, a baseline assessment should be established and followed by regular monitoring (e.g., every 4–6 weeks) throughout treatment and after discontinuation (59, 60). Prospective surveillance based on the known temporal patterns of toxicity is crucial for the early identification and management of irAEs, thereby preventing progression to severe events.

### 4.3 Demographic variables and irAEs

The results of binary logistic regression analysis revealed that patients aged <65 years faced a higher risk of developing irAEs, which was consistent with findings from several studies suggesting a greater irAE risk in younger patients (30, 61–63). This may be attributed to a more robust immunomodulatory function in younger individuals. However, this conclusion remains controversial. A systematic review reported conflicting associations between irAEs and age: nine studies identified a positive correlation between increasing age and irAE risk—potentially linked to the higher overall prevalence of comorbidities, cancer risk, and frailty in older adults—whereas seven studies found an inverse association. The contradictory relationship between age and irAE occurrence suggests that age alone may not serve as a reliable indicator of irAE risk; confounding factors, such as the use of less toxic treatment regimens in elderly patients, could further obscure this association (1).

To address the potential confounding effects of other demographic variables, we performed sensitivity analyses by adding sex, BMI, smoking history, and comorbidities to the models. The results showed that none of these variables were significantly and independently associated with irAEs or EirAEs (all P>0.05). A review noted that some studies have reported associations between an increased risk of irAEs and several patient-related factors, including higher BMI, men in PD-1/PD-L1, women in CTLA-4, smoking of ≥50 pack-years or current smoker, pre-existing comorbidities (for example, autoimmune disease, interstitial lung disease) (30). Certain irAE appear to be more frequently associated with specific cancer types (64). Gastrointestinal and dermatologic irAE may occur more commonly in patients with melanoma compared to NSCLC while higher prevalence of dermatitis, arthritis and myalgia may occur in patients with NSCLC compared to melanoma (65). Inconsistencies exist across different studies. The discrepancies may be attributed to several factors, including differences in study population, treatment regimen, cancer types and sample size. Notably, after adjusting for demographic variables, our findings remained robust.

### 4.4 Corticosteroids and irAEs

Analysis of concomitant medications showed that corticosteroid use emerged as a protective factor against irAEs, while PPI, NSAIDs, and antibiotics did not show statistically significance. The corticosteroids used were mostly dexamethasone at a dose of 5mg. Its protective effect may be related to the anti-inflammatory and immunosuppressive properties of corticosteroids. A retrospective review indicated that corticosteroid use prior to the initiation of immunotherapy [average dose of 35.2 ± 2.49mg (prednisone equivalent)] was associated with a lower incidence of irAEs (adjusted OR = 0.143, 95%CI:0.036-0.562, P = 0.005) (66). Early administration of these drugs was thought to potentially reduce the morbidity and mortality associated with severe irAEs, particularly those involving critical organs such as the heart and nervous system (67, 68). However, a multicenter retrospective study of patients with advanced or recurrent non-small cell lung cancer suggested that using a dexamethasone-equivalent dose of <15.9mg for prophylaxis against chemotherapy-induced nausea and vomiting may increase the risk of irAEs (69). Given the immunosuppressive effects of steroids, their potential impact on immunotherapy efficacy warrants consideration. The large phase III RCT studies KEYNOTE-189 and KEYNOTE-407 both found that short-term pretreatment with low-dose corticosteroids in patients receiving immunotherapy combined with chemotherapy did not significantly affect the efficacy of ICIs (17, 70). However, other studies have shown that immunotherapy efficacy was worse in patients receiving ≥10mg prednisone than in those receiving 0–10 mg and pointed out that differences in the efficacy of corticosteroid therapy may be due to specific factors related to the need for palliative steroid treatment, such as tumor burden and performance status (71, 72). A prospective cohort study demonstrated that patients who received corticosteroids before starting immunotherapy had significantly lower overall survival than non-users(HR = 1.37, P = 0.005), with systemic steroid use showing a stronger association with poorer survival (39). Current guidelines do not recommend their routine use prior to immunotherapy for adverse event prophylaxis, considering their potential negative impact on ICI efficacy and inherent side effects (73).

### 4.5 Combination therapy strategies and irAEs

The use of ICIs, particularly in combination with other agents, may increase the incidence of irAEs, potentially leading to novel and distinct patterns of irAEs (28, 74). This study found that although chemotherapy and targeted therapy did not show a significant impact on the overall incidence of irAEs, targeted therapy was significantly associated with the development of EirAEs. A meta-analysis showed that combining ICI therapy with targeted therapy, compared to non-combination targeted therapy, increased the risk of EirAEs by 2.71-fold (95%CI:2.11-3.47). Combination with chemotherapy (OR = 1.18, 95%CI:0.97-1.42) was not identified as a risk factor for EirAEs. Regarding specific mechanisms, literature reported that tyrosine kinase inhibitors, particularly those targeting vascular endothelial growth factor receptors (VEGFR) or platelet-derived growth factor receptors, could lead to thyroid dysfunction. VEGFR blockade could result in decreased blood perfusion and parenchymal ischemia, leading to hypothyroidism (75, 76). Other recently described mechanisms for Sunitinib-induced hypothyroidism include impairment of thyroid iodine uptake (77) and inhibition of thyroid peroxidase (78). Chemotherapy may activate Tregs, which function to suppress immune responses and maintain self-tolerance. The activation of Tregs could lead to immune suppression, complicating the relationship between the combination of ICIs and chemotherapy regarding the incidence of irAEs (79). Current guidelines for ICI−related practice also clearly indicated that combination chemotherapy is a risk factor for immune−related non−organ−specific adverse events (30). A study indicated that irAEs were more likely to occur when immunotherapy was combined with chemotherapy, targeted therapy, or both, compared to immune monotherapy (41). Additionally, chemotherapy and targeted therapy could independently induce various adverse events, which may resemble the clinical manifestations of irAEs, complicating the assessment of causality.

### 4.6 Predictive value of laboratory biomarkers

This study found that a higher baseline TSH level was a risk factor for EirAEs. A meta-analysis showed that for each 1 mU/L increase in baseline TSH, the risk of EirAEs was 1.3-fold greater (95%CI: 1.10-1.53) (75). Multiple studies have also indicated that patients with elevated baseline TSH face a significantly higher risk of developing primary hypothyroidism (49, 80). Given that TSH testing is simple and readily accessible, it can serve as an effective tool in clinical practice for predicting endocrine toxicity. Relevant guidelines from the American Society of Clinical Oncology (ASCO) also recommended routine monitoring of TSH (optionally combined with free T4) in patients receiving immunotherapy, with repeat testing every 4–6 weeks, to enable early identification and management of thyroid-related irAEs (81).

A systematic review reported that among 18 studies that evaluated predictive models for irAEs, 13 assessed laboratory risk factors, including serum cytokines, blood cell ratios, and TSH. However, evidence remains heterogeneous due to variations in study design, ICI agents used, risk factors investigated, method used to detect and measure irAE, and method employed to analyze the association between factors and irAE occurrence (1). A review summarized that ALC, AEC, platelet count, NLR, and PLR were associated with irAE risk. The review also summarized that higher baseline TSH (>1.67 mIU/L) and detection of anti-thyroid peroxidase or anti-thyroglobulin antibodies predicted thyroid irAE. Furthermore, lower baseline levels of TNF-α, IL-6, IL-8, CXCL9, CXCL10, and CXCL11 were associated with higher irAE risk, while a post-treatment rise in IL-6, CXCL5, CXCL9, and CXCL10 indicated impending irAE (30). A study reported that in 418 cancer patients treated with anti-PD-1/PD-L1 inhibitors, higher post-treatment NLR (OR = 1.454, P = 0.024), and higher baseline circulating tumor cell (CTC) level (OR = 1.104, P = 0.013) were independent predictors of irAE occurrence. In addition, lower baseline prognostic nutritional index (PNI, P = 0.048), and higher post-treatment LDH level (P = 0.031) were associated with more severe irAEs (82). This study did not identify predictive value for NLR, inflammatory cytokines (IL-6, IL-8, TNF-α), or proBNP. Further validation with larger sample sizes and more rigorous study designs is warranted. The predictive value of other potential biomarkers, such as CTCs, PNI, eosinophils, C-reactive protein, HLA genes, microRNAs, gene expression profiling, and intestinal microbiome, also requires further validation in the context of combination therapy.

## 5 Limitations

The limitations of this study are primarily reflected in the following aspects: Firstly, the retrospective single-center design carries inherent selection and information biases, and the overall limited sample size may affect the reliability of the results. Although sensitivity analyses were performed, the possibility of residual confounding cannot be completely excluded. Secondly, data collection based on the hospital information system may lead to under-reporting or oversight of mild irAEs. Thirdly, due to loss to follow-up or incomplete medical records for some patients, it was not possible to further explore the association between treatment efficacy and irAEs. Finally, although multiple imputation was employed to handle missing data, this may still introduce some biases.

## 6 Conclusions

This study demonstrated that among Chinese patients predominantly treated with ICI combination therapy, the overall incidence of irAEs was 38.05%, primarily grade 1–2 adverse events. Corticosteroid use was a protective factor against irAEs, and age≥65 years was associated with lower risk. Combination with targeted therapy and higher baseline TSH levels were risk factors for EirAEs. Furthermore, the timing of onset differed among toxicities, with the majority occurring within the first 4 treatment cycles. These findings suggest that individualized monitoring strategies should be implemented in clinical practice based on different risk factors and the temporal patterns of toxicities. Future prospective, multicenter studies are warranted, incorporating deeper biological exploration (e.g., dynamic cytokine profiling, gut microbiome, autoantibodies) to better distinguish the sources of toxicity in combination therapy and to analyze the relationship between irAEs and treatment efficacy.

## Data Availability

The raw data supporting the conclusions of this article will be made available by the authors, without undue reservation.
