# Supplementary material for: Characteristics and risk factors of immune-related adverse events in patients receiving immune checkpoint inhibitor combination therapy
Source: Front Oncol. 2026 May 29;16:1808832. doi: 10.3389/fonc.2026.1808832 (PMC13259756; doi:10.3389/fonc.2026.1808832)
Supplement: Supplementary file 1 [file DataSheet1.docx]

Supplementary Table S1. Missing variables and imputation.

| Characteristics | Missing, n | Missing(%) | Imputed |
| --- | --- | --- | --- |
| proBNP | 69 | 23.23 | * |
| LDH | 49 | 16.50 | * |
| IL-6 | 49 | 16.50 | * |
| IL-8 | 47 | 15.82 | * |
| TNF-α | 46 | 15.49 | * |
| T3 | 37 | 12.46 | * |
| T4 | 37 | 12.46 | * |
| TSH | 37 | 12.46 | * |
| NLR | 30 | 10.10 | * |

*imputed. proBNP:pro-brain natriuretic peptide; LDH:lactate dehydrogenase; IL-6:interleukin-6; IL-8:interleukin-8; TNF-α:tumor necrosis factor-alpha; T3:triiodothyronine; T4:thyroxine; TSH:thyroid-stimulating hormone; NLR:neutrophil-to-lymphocyte ratio.

Supplementary Table S2. Distribution of irAEs across different ICIs(N=297).

| Type of irAEs | Sintilimab | Tislelizumab | Camerlizumab | Adebrelimab | Toripalimab | Cadonilimab | Serplulimab | Atezolizumab | Penpulimab | N(%) |
| --- | --- | --- | --- | --- | --- | --- | --- | --- | --- | --- |
| Endocrine toxicity | 16 | 8 | 6 | 4 | 2 | 0 | 0 | 0 | 0 | 36(12.12%) |
| Gastrointestinal Toxicity | 10 | 4 | 10 | 1 | 0 | 0 | 1 | 0 | 0 | 26(8.75%) |
| Dermatologic Toxicity | 10 | 3 | 2 | 1 | 0 | 1 | 1 | 1 | 1 | 20(6.73%) |
| Hepatic toxicity | 5 | 6 | 2 | 0 | 1 | 0 | 0 | 0 | 0 | 14(4.71%) |
| Hematologic Toxicity | 6 | 2 | 0 | 2 | 0 | 1 | 0 | 0 | 0 | 11(3.70%) |
| Neurologic toxicity | 3 | 1 | 1 | 0 | 1 | 0 | 0 | 0 | 0 | 6(2.02%) |
| Cardiac toxicity | 0 | 2 | 2 | 0 | 0 | 0 | 0 | 0 | 0 | 4(1.35%) |
| Lung toxicity | 1 | 1 | 1 | 0 | 0 | 0 | 0 | 0 | 0 | 3(1.01%) |
| Renal toxicity | 1 | 0 | 1 | 0 | 0 | 0 | 0 | 0 | 0 | 2(0.67%) |

irAEs: immune-related adverse events; ICIs: immune checkpoint inhibitors.

Supplementary Table S3. Distribution of irAEs and EirAEs across different cancer types.

| Cancer types(n) | irAEs(n,%) | EirAEs(n,%) |
| --- | --- | --- |
| Stomach cancer(61) | 24(39.34%) | 5(8.20%) |
| Lung cancer(47) | 16(34.04%) | 1(2.13%) |
| Gynecological cancer(39) | 16(41.03%) | 4(10.26%) |
| Soft tissue cancer(37) | 11(29.73%) | 8(21.62%) |
| Other cancers(30) | 13(43.33%) | 6(20.00%) |
| Bone cancer(24) | 10(41.67%) | 5(20.83%) |
| Urinary system cancer(17) | 7(41.18%) | 3(17.65%) |
| Colorectal cancer(17) | 9(52.94%) | 1(5.88%) |
| Head and neck cancer(15) | 6(40.00%) | 3(20.00%) |
| Esophageal cancer(10) | 1(10.00%) | 0 (0.00%) |

irAEs: immune-related adverse events; EirAEs: Endocrine immune-related adverse events.

Supplementary Table S4. Lasso regression variable screening for EirAEs.

| Characteristics | N (%) |
| --- | --- |
| Corticosteroids(Yes) | 46 (92.0%) |
| TSH | 43 (86.0%) |
| Chemotherapy(Yes) | 43 (86.0%) |
| Targeted therapy(Yes) | 34 (68.0%) |
| NLR | 29 (58.0%) |
| Age(≥65) | 14 (28.0%) |

λ1se selected as the optimal value. The percentage represents the proportion of variables that were selected among the 50 imputed datasets. EirAEs: Endocrine immune-related adverse events; TSH: thyroid-stimulating hormone; NLR: neutrophil-to-lymphocyte ratio.

Supplementary Table S5. Sensitivity analysis of binary logistic regression for irAEs.

| Characteristics | Coefficient | Wald | OR | 95% CI | P value |
| --- | --- | --- | --- | --- | --- |
| Intercept | -0.699 | -0.831 | 0.497 | (0.095-2.602) | 0.407 |
| Chemotherapy |  |  |  |  |  |
| Yes(vs No) | -0.276 | -0.832 | 0.759 | (0.395-1.458) | 0.406 |
| Corticosteroids |  |  |  |  |  |
| Yes(vs No) | -0.674 | -2.4 | 0.509 | (0.293-0.886) | **0.017** |
| Age |  |  |  |  |  |
| ≥65(vs＜65) | -0.716 | -2.631 | 0.489 | (0.286-0.835) | **0.009** |
| TSH | 0.013 | 0.869 | 1.013 | (0.984-1.044) | 0.386 |
| Sex |  |  |  |  |  |
| Female(vs Male) | 0.041 | 0.149 | 1.041 | (0.61-1.777) | 0.881 |
| Smoking history |  |  |  |  |  |
| Yes(vs No) | -0.045 | -0.124 | 0.956 | (0.471-1.944) | 0.902 |
| BMI | 0.044 | 1.366 | 1.045 | (0.981-1.115) | 0.173 |
| Comorbidities |  |  |  |  |  |
| Yes(vs No) | 0.09 | 0.335 | 1.094 | (0.645-1.855) | 0.738 |

The binary logistic regression analysis was adjusted for demographic variables, including Sex, Smoking history, BMI, and Comorbidities. irAEs: immune-related adverse events; OR: odd ratio; TSH: thyroid-stimulating hormone; BMI:body mass index. Significant values are in [bold].

Supplementary Table S6. Sensitivity analysis of binary logistic regression for EirAEs.

| Characteristics | Coefficient | Wald | OR | 95% CI | P value |
| --- | --- | --- | --- | --- | --- |
| Intercept | -2.377 | -1.839 | 0.093 | (0.007-1.182) | 0.067 |
| Targeted therapy |  |  |  |  |  |
| Yes(vs No) | 1.113 | 2.388 | 3.044 | (1.216-7.62) | **0.018** |
| Corticosteroids |  |  |  |  |  |
| Yes(vs No) | -1.518 | -3.179 | 0.219 | (0.086-0.561) | **0.002** |
| Chemotherapy |  |  |  |  |  |
| Yes(vs No) | -0.491 | -0.978 | 0.612 | (0.228-1.644) | 0.329 |
| TSH | 0.033 | 2.086 | 1.033 | (1.002-1.066) | **0.038** |
| Age |  |  |  |  |  |
| ≥65(vs＜65) | -0.859 | -1.838 | 0.423 | (0.169-1.063) | 0.067 |
| Sex |  |  |  |  |  |
| Female(vs Male) | 0.702 | 1.62 | 2.017 | (0.86-4.731) | 0.106 |
| Smoking history |  |  |  |  |  |
| Yes(vs No) | 0.215 | 0.353 | 1.24 | (0.373-4.121) | 0.724 |
| BMI | 0.018 | 0.368 | 1.018 | (0.925-1.121) | 0.713 |
| Comorbidities |  |  |  |  |  |
| Yes(vs No) | 0.521 | 1.205 | 1.684 | (0.719-3.945) | 0.229 |

The binary logistic regression analysis was adjusted for demographic variables, including Age, Sex, Smoking history, BMI, and Comorbidities. EirAEs: Endocrine immune-related adverse events; OR: odd ratio; TSH: thyroid-stimulating hormone; BMI:body mass index. Significant values are in [bold].

Supplementary Table S7. Binary logistic regression analysis of irAEs in the complete dataset.

| Characteristics | Coefficient | Wald | OR | 95% CI | P value |
| --- | --- | --- | --- | --- | --- |
| Intercept | 0.274 | 0.85 | 1.315 | (0.698-2.487) | 0.395 |
| Chemotherapy |  |  |  |  |  |
| Yes(vs No) | -0.179 | -0.522 | 0.836 | (0.427-1.651) | 0.602 |
| Corticosteroids |  |  |  |  |  |
| Yes(vs No) | -0.722 | -2.456 | 0.486 | (0.272-0.863) | 0.014 |
| Age |  |  |  |  |  |
| ≥65(vs＜65) | -0.797 | -2.837 | 0.451 | (0.257-0.776) | 0.005 |
| TSH | 0.010 | 0.691 | 1.010 | (0.984-1.045) | 0.490 |

The Wald statistic is used to test the significance of individual variables in the model. irAEs: immune-related adverse events; OR: odd ratio; TSH: thyroid-stimulating hormone.

Supplementary Table S8. Binary logistic regression analysis of EirAEs in the complete dataset.

| Characteristics | Coefficient | Wald | OR | 95% CI | P value |
| --- | --- | --- | --- | --- | --- |
| Intercept | -2.207 | -4.055 | 0.110 | (0.036-0.303) | <0.001 |
| Targeted therapy |  |  |  |  |  |
| Yes(vs No) | 1.464 | 2.976 | 4.322 | (1.695-11.873) | 0.003 |
| Corticosteroids |  |  |  |  |  |
| Yes(vs No) | -1.641 | -3.254 | 0.194 | (0.069-0.505) | 0.001 |
| Chemotherapy |  |  |  |  |  |
| Yes(vs No) | 0.122 | 0.230 | 1.130 | (0.406-3.301) | 0.818 |
| TSH | 0.033 | 2.143 | 1.034 | (1.005-1.075) | 0.032 |

The Wald statistic is used to test the significance of individual variables in the model. EirAEs: Endocrine immune-related adverse events; OR: odd ratio; TSH: thyroid-stimulating hormone.
